# Hydroxychloroquine Prevents High-altitude Cerebral Edema by Inhibiting Endothelial Claudin-5 Autophagic Degradation

**DOI:** 10.2174/011570159X371235250417051313

**Published:** 2025-05-09

**Authors:** Yan Xue, Baolan Wan, Zhen Wang, Zhiwei Wang, Dongzhi Wang, Wanping Yang, Xueting Wang, Li Zhu

**Affiliations:** 1 Research Center of Molecular Medicine, Nantong Health College of Jiangsu Province, Nantong, 226010, China;; 2 Institute of Special Environmental Medicine, Co-Innovation Center of Neuroregeneration, Nantong University, Nantong, 226019, China;; 3 Department of Hepatobiliary and Pancreatic Surgery, Affiliated Hospital of Nantong University, Nantong, 226006, China

**Keywords:** Hydroxychloroquine, high-altitude cerebral edema, Claudin-5, autophagy, blood-brain barrier, prolonged hypobaric hypoxia

## Abstract

**Background:**

High-altitude cerebral edema (HACE) is a serious condition caused by prolonged hypobaric hypoxia (HH). Autophagic degradation of Claudin-5 plays a crucial role in HH-induced blood-brain barrier (BBB) damage. Hydroxychloroquine (HCQ), a lysosomal inhibitor used in autophagy treatment, reduces inflammation and BBB damage in traumatic brain injury. However, its effectiveness in preventing HACE is still unknown.

**Methods:**

C57BL/6J mice were treated with HCQ and exposed to HH for 24 hrs to study BBB integrity. We evaluated BBB disruption *via* brain water content, Evans blue, and FITC-dextran assays. Changes in tight junctions (TJs) of cerebrovascular endothelial cells were analyzed using electron microscopy and immunofluorescence. Western blotting quantified autophagy protein levels in brain tissue. Hypoxia-mimetic *in vitro* models were used to explore HCQ's effects on TJs and BBB permeability, confirmed by various assays, including immunofluorescence, electron microscopy, and Western blotting.

**Results:**

HCQ significantly mitigated rapamycin-induced autophagy and Claudin-5 degradation. Prolonged hypoxia exposure promoted lysosomal degradation of Claudin-5, increasing endothelial cell permeability. HCQ inhibited autophagy in bEnd.3 cells *via* the PI3K-Akt-mTOR and Erk pathway, reducing hypoxia-induced Claudin-5 down-regulation. In mice, HH exposure increased brain autophagy, damaging the vascular endothelial TJs and subsequently increasing endothelial permeability. Pretreatment with HCQ significantly reduced the level of autophagy in the brains of HH-exposed mice, thereby mitigating the HH-induced damage to vascular TJs, alleviating the downregulation of Claudin-5, and enhancing endothelial integrity.

**Conclusion:**

HCQ effectively prevented HACE by inhibiting HH-induced Claudin-5 membrane expression downregulation, thus mitigating BBB damage and brain water content increase in HH-exposed mice.

## 1 INTRODUCTION

High-altitude cerebral edema (HACE) is a life-threatening condition that arises from rapid ascent to high altitudes, with an incidence rate of 0.5-1.0% among populations residing at altitudes of 4,000-5,000 m annually [1]. This incidence is on the rise due to the increasing popularity of high-altitude tourism [2]. HACE is widely acknowledged as the advanced stage of acute mountain sickness, marked by severe fatigue, ataxia, and neuropsychiatric symptoms, which can escalate to significant cognitive dysfunction, somnolence, coma, and brain herniation [3, 4]. If not promptly diagnosed and treated, HACE can swiftly progress to coma and death within 24 hrs due to brain herniation [5]. Given the challenges posed by the alpine environment, effectively treating HACE is difficult, underscoring the importance of prevention.

However, there are currently no clinical drugs available for the prevention of HACE. The most effective treatments are oxygen therapy and descent to lower altitudes [6, 7]. Due to various objective constraints, patients with HACE often endanger their lives by not receiving timely and effective first aid. Recent research has focused on elucidating how different pharmacological therapies affect the integrity and function of the blood-brain barrier (BBB) in the context of HACE. Portable hyperbaric oxygen chambers are used as an alternative, supplemented with symptomatic treatments such as dexamethasone, acetazolamide, diuretics, mannitol, and hypertonic saline to reduce intracranial pressure and enhance cerebral circulation [8, 9]. Studies have demonstrated that dexamethasone decreases the expression of pro-inflammatory cytokines, which are known to compromise the integrity of tight junctions within the BBB [10]. In animal models, the administration of mannitol has resulted in reduced edema and improved neurological outcomes, correlating with enhanced preservation of BBB integrity [11]. Additionally, the use of antioxidants, such as N-acetylcysteine, has been shown to mitigate oxidative damage to the BBB. This restoration of antioxidant capacity is associated with improved BBB function, as evidenced by decreased permeability in HACE models [12]. While existing clinical drugs like dexamethasone and acetazolamide are effective in preventing and treating HACE, they all carry significant contraindications or adverse effects. Dehydration or excessive diuresis can cause severe complications. As the number of individuals exposed to high altitudes steadily rises, the identification of safe and efficacious medications to treat HACE becomes imperative.

Understanding the underlying pathophysiology of HACE is crucial in guiding effective treatment strategies. Neuroimaging of patients with HACE reveals angiogenic edema with microhemorrhages in the white matter and corpus callosum [13, 14]. Vascular edema, resulting from BBB damage and plasma protein leakage into the extracellular matrix, leading to perivascular and intercellular accumulation, is considered the primary pathophysiological mechanism of HACE [15]. This accumulation results in severe brain tissue swelling and potentially fatal brain herniation. The BBB is composed of continuous capillary endothelium, pericytes, and astrocyte end feet, with the intercellular tight junctions (TJs) formed by the endothelium being the critical component [16]. Hypoxia-induced disruption of endothelial TJs leads to BBB injury and vasogenic edema, the primary cause of HACE [17, 18]. Claudin-5, a key tight junction protein expressed in cerebrovascular endothelium, is vital for maintaining BBB integrity [19, 20]. Previous research indicates that hypobaric hypoxia (HH) exposure damages the BBB and induces HACE by promoting autophagic degradation of Claudin-5 [18]. Upregulation of Claudin-5 presents novel drug targets and pathways to modulate BBB permeability in treating central nervous system diseases [21, 22]. Therefore, inhibiting the autophagic degradation of Claudin-5 could be a potential therapeutic strategy for HACE.

In this context, hydroxychloroquine (HCQ) emerges as a promising candidate due to its ability to modulate autophagic processes. HCQ, classified under 4-aminoquinolines, features a flat aromatic core structure and exhibits weak basicity due to its basic side chain. This side chain facilitates the accumulation of the drug in intracellular compartments, particularly lysosomes, which is crucial for its activity and potential interaction with nucleic acids. Lysosomal channels guide the catabolic “self-eating” process named autophagy, which is mainly involved in protein and organelle quality control [23]. HCQ is extensively used for the prevention and treatment of malaria [24, 25], as well as for systemic lupus erythematosus, reducing the incidence of cardiovascular diseases while also improving survival in rheumatoid arthritis by alleviating insulin resistance [26]. The Food and Drug Administration has approved HCQ for treating rheumatic and skin diseases, and it is currently undergoing clinical trials for potential applications in Corona Virus Disease 2019, various cancers, type I and II diabetes, multiple sclerosis, recurrent abortion, and myocardial infarction [27-30]. HCQ is renowned for its rapid onset, prolonged action, low toxicity, and high tolerability in humans. Notably, HCQ has been reported to cross the BBB [31]. At the molecular level, HCQ interferes with lysosomal activity and autophagy [32], leading to the hypothesis that it could target the autophagic degradation of Claudin-5 in cerebrovascular endothelial cells.

This study aims to explore whether HCQ mitigates the lysosomal degradation of Claudin-5, thereby reducing tight junction destruction and BBB injury induced by HH, ultimately preventing HACE. This research endeavors to establish a theoretical foundation for the early treatment and prevention of HACE.

## 2 MATERIALS AND METHODS

### 2.1 Mice Grouping

Male C57BL/6 mice, aged 8 weeks (n=12), were obtained from the Experimental Animal Centre of Nantong University. The mice, with body weights ranging from 20 ± 2 g, were weighed and sequentially numbered from the lightest to the heaviest. Mice that do not meet the specified body weight criteria will be excluded from the experiment. Subsequently, they were assigned to groups using a random number table. Then, the mice were randomly assigned to one of the following groups: Control group, mice treated with HH, mice treated with HH + HCQ (60 mg/kg), and group housed in polycarbonate cages (four per cage of 30 ×30 × 15 cm). The mice were kept under a 12/12 hrs light-dark cycle at a temperature of 22 ± 1.5^o^C. Water and food pellets are available ad libitum.

### 2.2 HCQ Preparation and Treatment

#### 2.2.1 HCQ Preparation

HCQ (M.W. = 335.87, CAS 747-36-4) with a purity of 98% was procured from Merck. A 30 mM stock solution was prepared by dissolving 30 mg of HCQ powder in 2.9773 mL of DMSO. The mixture was sonicated for 5 s three times to aid dissolution, aliquoted, and stored at -80°C. For cellular treatments, the stock solution was diluted with Dulbecco’s Modified Eagle Medium (DMEM) containing 10% serum to obtain a 30 μM working solution. For animal treatments, the HCQ stock was diluted in saline. C57BL/6 mice received a single dose of 30 mg/kg HCQ *via* intraperitoneal injection [33], administered twice before HH exposure. The control group mice received 200 μL of saline.

#### 2.2.2 HCQ Treatment of bEnd.3 Cells

bEnd.3 cells (Bioleaf Biotech), a mouse brain microvascular endothelial cell line, were cultured in DMEM supplemented with 10% heat-inactivated fetal bovine serum, 4.5 g/L glucose, 1% glutamine, and 1% penicillin/streptomycin at 37°C in a humidified atmosphere containing 5% CO_2_. For HCQ treatment, cells were pre-incubated with 30 μM HCQ for 2 hrs, followed by exposure to hypoxia (94% N_2_, 5% CO_2_, and 1% O_2_) [34-36] or 50 nM rapamycin for 24 hrs with continuous HCQ administration.

#### 2.2.3 HCQ Treatment of Animals

For HCQ treatment, mice were intraperitoneally injected with HCQ (30 mg/kg) twice daily to inhibit autophagy before exposure to HH [33]. The study was conducted following the guidelines of the Nantong University Institutional Animal Care and Use Committee for animal care, handling, and experimental procedures (S20220219-007).

### 2.3 HACE Model Construction and Determination

#### 2.3.1 HACE Model Construction

Mice were subjected to an altitude of 7,000 m in a decompression chamber at a rate of 300 m/min for 48 hrs, followed by the descent to sea level at the same speed [37-39]. For normobaric normoxia (NN) treatment, mice were kept outside the chamber in the same laboratory setting. After HH exposure, the mice were anesthetized and perfused with physiological saline (0.9%) to remove the blood.

For each animal, three different investigators were involved as follows: the first investigator administered the treatment based on the randomization table. This investigator was the only person aware of the treatment group allocation. A second investigator was responsible for the anaesthetic procedure and the surgical procedure. Finally, a third investigator (also unaware of treatment) assessed the primary outcome measure.

#### 2.3.2 Brain Water Content Assay

Mouse brains were collected under 1% sodium pentobarbital anesthesia and immediately weighed using a precision electronic balance. The brains were then dried at 100°C until a constant weight was achieved. Brain water content was calculated using the formula: brain water content (%) = (wet weight - dry weight) / wet weight × 100%.

#### 2.3.3 BBB Permeability Assay

BBB permeability was assessed by measuring the extravasation of Evans blue (EB, Sigma Aldrich, CAS: 314-13-6). Mice were injected with EB dye (2%, 4 mL/kg) *via* the tail vein following HH exposure and allowed to circulate for 1 h. Mice were then transcardially perfused with physiological saline (0.9%) through the left ventricle to remove any remaining intravascular dye. One-half of the brain was weighed, homogenized, and placed in polyethylene tubes containing 1 mL of 50% trichloroacetic acid solution formamide. The supernatants were collected and diluted with ethanol at a 1:3 ratio, and their absorbance was measured at 620 nm using a plate reader (Synergy 2TM, Bio-Tek, US). A standard curve was established to determine the dye quantity, expressed as micrograms per gram (µg/g) of brain tissue. The other half of the brain was fixed and sectioned for confocal imaging using a 647 nm laser on a Leica SP8 confocal microscope.

### 2.4 Determination of Hypoxia-exposed Endothelial Cells

#### 2.4.1 Transendothelial Electrical Resistance (TEER)

The integrity of tight junctions enhances TEER, whereas BBB impairment results in decreased electrical resistance of the cell monolayer [40]. To evaluate the effect of hypoxia on BBB integrity, TEER measurements were performed using a Millicell ERS-2 Epithelial Volt-ohm Meter (Merck Millipore) on bEnd.3 cells were cultured on transwell inserts (3401 Corning) until confluence. Following calibration, the long arm of the electrode was placed in the basolateral culture medium and the short arm in the apical medium, ensuring no contact with the cell layer or insert wall. TEER was calculated using the formula: TEER (Ω·cm^2^) = (total resistance - blank resistance) (Ω) × insert area (cm^2^).

#### 2.4.2 Transcytosis Assay

An endothelial monolayer with intact tight junctions restricts dextran permeation from the upper to lower chamber, whereas tight junction disruption allows dextran leakage, reflecting impaired barrier integrity. The concentration of dextran in the lower chamber measures endothelial barrier function [41]. bEnd.3 cells were cultured on transwell inserts (3401, Corning) to form a complete endothelial fusion layer. Subsequently, 100 μg/mL of FITC-dextran 40 kDa was added to the inserts for 30 mins. Enhanced permeability of the fusion layer allows FITC-dextran to penetrate the lower medium. The concentration of FITC-dextran in the lower chambers was measured using a microplate reader at Ex490 nm/ Em520 nm.

#### 2.4.3 Transmission Electron Microscopy (TEM)

Brain sections and bEnd.3 cells were fixed with 4% glutaraldehyde in phosphate buffer and stored overnight at 4°C. After washing with PBS, brain sections and the cells were dehydrated in graded ethanol (50%, 75%, and 95%) and embedded in epoxy resin. Ultrathin sections were cut to a thickness of 70 nm and stained with uranyl acetate and lead citrate. Representative areas of the sections were analyzed using a TEM (HT7700, HITACHI, Japan), and the autophagic vacuoles in single cells were counted.

### 2.5 Immunofluorescence

Brain sections (40 µm thick) and cultured cells were permeabilized using 0.3% Triton X-100 and blocked with 10% donkey serum for 1 h. Samples were incubated overnight at 4°C with primary antibodies, including anti-Claudin-5 (Thermo, 35-2500), anti-Laminin (Abcam, ab11575), or anti-LAMP1 (RD, AF4320-SP). After washing, samples were incubated for 1 h at 37°C with secondary antibodies such as Alexa Fluor 555-conjugated donkey anti-rabbit IgG (Thermo, A31572), Alexa Fluor 647-conjugated donkey anti-mouse IgG (Thermo, A32787), or Alexa Fluor 488-conjugated donkey anti-goat IgG (Abcam, ab150133). Finally, DAPI (2 μg/mL) staining was performed in the dark for 10 mins, and imaging was conducted using a Leica SP8 confocal microscope.

### 2.6 Protein Isolation and Western Blot

Tissue and cell samples were lysed in radioimmunoprecipitation assay (RIPA) buffer containing 1% phenylmethanesulfonyl fluoride (PMSF) protease inhibitor and 1% phosphatase inhibitor. After thorough homogenization, supernatants were collected as soluble protein *via* centrifugation at 12,000 × g and 4°C for 15 mins. The insoluble material was lysed in RIPA buffer containing 20% sodium dodecyl sulfate, 24 M urea, and 1% PMSF protease inhibitor, followed by centrifugation. The resulting supernatant was collected as an insoluble protein. Proteins were denatured, transferred to a PVDF membrane, and blocked with 5% nonfat milk. Membranes were then incubated overnight at 4°C with the following antibodies: Claudin-5 (Thermo, 35-2500), LAMP1 (RD, AF4320-SP), p62 (Proteintech, 18420-1-AP), LC3 (CST, 12741S), ATP1A1 (Abcam, ab300507), mTOR (CST, 2983), p-mTOR (CST, 5536), PI3K (CST, 4257), p-PI3K (CST, 4228), Akt (CST, 4691), p-Akt (CST, 4060), Erk (Abcam, ab184699), p-Erk (Abcam, ab201015), and anti-β-actin (Sigma, A5316). Membranes were subsequently incubated with goat anti-rabbit HRP-conjugated secondary antibody (Jason, 115-035-033) or goat anti-mouse HRP-conjugated secondary antibody (Jason, 111-035-003).

### 2.7 RNA Isolation and Quantitative Real-time PCR

Total RNA was extracted from cells using Trizol. One microgram of total RNA was reverse transcribed into cDNA utilizing the HiScript^®^ III RT SuperMix kit (Vazyme, R323-01). The resulting cDNA underwent qRT-PCR with the SYBR qPCR Master Mix kit (Roche, USA). Relative mRNA expression levels of target genes were calculated using the ΔCt method. The primer sequences were as follows: *Lamp1* forward: 5′-CAGCACTCTTTGAGGTGAAAAAC-3′, reverse: 5′-ACGATCTGAGAACCATTCGCA-3′′; *Actb* forward: 5′-CATCCGTAAAGACCTCTATGCCAAC-3′, reverse: 5′-ATGGAGCCACCGATCCACA-3′.

### 2.8 Cyto-ID Autophagy Detection Kit Assay

To evaluate autophagy in living cells, a transfection-free kit for rapid quantification was employed [27]. Cells at 70-80% confluence were pre-incubated with 30 μM HCQ for 2 hrs, followed by exposure to hypoxia. Following washes, Cyto-ID autophagy detection stain (Enzo) was applied for 30 mins in phenol-free DMEM at 37°C [42]. Hoechst counterstaining followed, and samples were analyzed using a Leica SP8 confocal microscope.

### 2.9 Statistical Analysis

Statistical analyses were conducted using GraphPad Prism software version 8.0 (GraphPad Software, San Diego, CA). All quantitative data were presented as mean ± SD. Data analysis involved unpaired t-test, one-way ANOVA, or two-way ANOVA followed by Tukey’s multiple comparisons test. A *P-*value of less than 0.05 was considered statistically significant. Significance values were denoted as follows: *P < 0.05, **P < 0.01, and ***P < 0.001, with n.s indicating no significance.

## 3 RESULTS

### 3.1 HCQ Alleviates Autophagic Degradation of Claudin-5 in Endothelial Cells

**Fig. (1):**
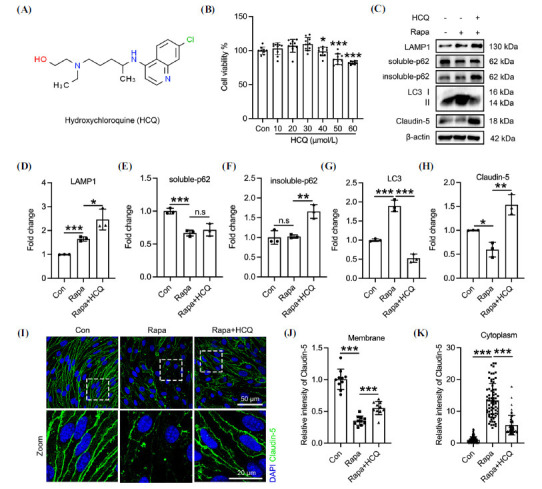
HCQ relieves autophagic degradation of Claudin-5 in endothelial cells. (**A**) Chemical structure and molecular formula of HCQ. (**B**) bEnd.3 cells were pretreated with HCQ for 24 hrs as indicated, and cell viability was measured using the CCK-8 assay. control 1.00 +/- 5.37, n=9; treatment 10 μM HCQ 102.96 +/- 8.79, n=10; treatment 20 μM HCQ 106.99 +/- 9.38, n=10; treatment 30 μM HCQ 109.59 +/- 9.49, n=10; treatment 40 μM HCQ 98.69 +/- 6.47, n=10, *p<*0.05; treatment 50 μM HCQ 87.38 +/- 7.77, n=10, *p<*0.001; treatment 60 μM HCQ 81.95 +/- 2.29, n=10, *p<*0.001. bEnd.3 cells were co-treated with 30 μM HCQ and 50 nM rapamycin (Rapa) for 24 hrs. (**C**) LAMP1, soluble-p62, insoluble-p62, LC3, and Claudin-5 were detected by Western blot. (**D** to **H**) Quantification of the grayscale values of LAMP1 (**D**) (control 1.00 +/- 0.006, n=3; treatment 50 nM rapamycin 1.65 +/- 0.092, n=3, *p<*0.001; treatment 30 μM HCQ and 50 nM rapamycin 2.46 +/- 0.437, n=3, *p<*0.05); soluble p62 (**E**) (control 1.00 +/- 0.043, n=3; treatment 50 nM rapamycin 0.67 +/- 0.045, n=3, *p<*0.001; treatment 30 μM HCQ and 50 nM rapamycin 0.72 +/- 0.094, n=3); insoluble p62 (**F**) (control 1.00 +/- 0.171, n=3; treatment 50 nM rapamycin 1.025 +/- 0.044, n=3; treatment 30 μM HCQ and 50 nM rapamycin 1.66 +/- 0.174, n=3, *p<*0.01); LC3 (**G**) (control 1.00 +/- 0.034, n=3; treatment 50 nM rapamycin 1.89 +/-0.151, n=3, *p<*0.01; treatment 30 μM HCQ and 50 nM rapamycin 0.53 +/- 0.109, n=3, *p<*0.01); and Claudin-5 (**H**) (control 1.00 +/- 0.005, n=3; treatment 50 nM rapamycin 0.59 +/- 0.153, n=3, *p<*0.05; treatment 30 μM HCQ and 50 nM rapamycin 1.54 +/- 0.213, n=3, *p<*0.01) in panel **C**. (**I**) bEnd.3 cells were fixed and stained with an anti-Claudin-5 antibody, followed by confocal imaging. (**J** and **K**) Quantification of Claudin-5 intensity in the plasma membrane (**J**) (control 1.00 +/- 0.160, n=10; treatment 50 nM rapamycin 0.355 +/- 0.072, n=10, *p<*0.001; treatment 30 μM HCQ and 50 nM rapamycin 0.55 +/- 0.103, n=10, *p<*0.001) and cytoplasm (**K**) (control 1.00 +/- 0.955, n=133; treatment 50 nM rapamycin 13.43 +/-5.349, n=71, *p<*0.001; treatment 30 μM HCQ and 50 nM rapamycin 5.66 +/- 3.102, n=73, *p<*0.001) of bEnd.3 cells in panel I. Significance values were denoted as follows: *p<0.05, **p<0.01, and ***p<0.001, with n.s indicating no significance.

Treatment of bEnd.3 cells with HCQ (Fig. **1A**) revealed that concentrations of 40 μM and above significantly reduced cell viability, while concentrations below 30 μM exhibited no apparent toxic effects (Fig. **1B**). Consequently, 30 μM HCQ was established as the safe concentration for subsequent experiments. To assess whether HCQ eases autophagy in endothelial cells, autophagy was first induced using rapamycin, followed by HCQ treatment. Rapamycin significantly upregulated LC3 and reduced soluble p62 levels in bEnd.3 cells. In contrast, HCQ inhibited autophagy, indicated by the downregulation of LC3 and upregulation of the lysosomal marker LAMP1 and insoluble p62 (Figs. **1C**-**1G**), suggesting HCQ's inhibitory effect on autophagy in endothelial cells. Given that Claudin-5 undergoes degradation *via* autophagy, rapamycin treatment was shown to downregulate Claudin-5 in endothelial cells, whereas HCQ prevented Claudin-5 degradation (Figs. **1C** and **H**). Immunofluorescence analysis further confirmed that HCQ not only enhanced Claudin-5 membrane localization but also significantly reduced its abnormal cytoplasmic accumulation in rapamycin-treated bEnd.3 cells (Figs. **1I**-**K**). These findings indicate that HCQ effectively alleviates autophagy, thereby preventing Claudin-5 degradation in endothelial cells.

### 3.2 Hypoxia Reduces the Membrane Expression of Claudin-5 in Vascular Endothelial Cells by Enhancing Autophagy

**Fig. (2):**
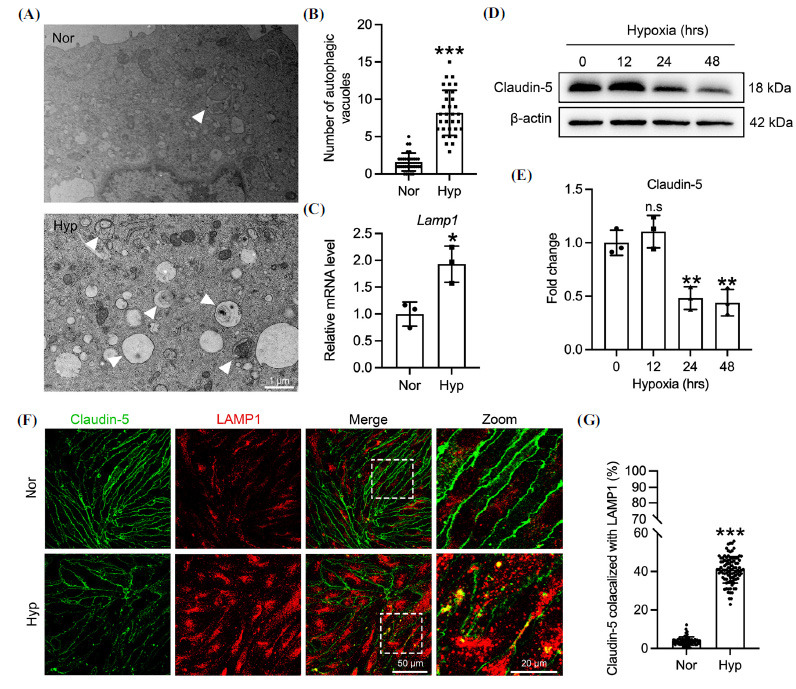
Hypoxia triggers the upregulation of autophagy and the downregulation of Claudin-5 in bEnd.3 cells. (**A**) Ultrastructure of bEnd.3 cells were treated with 1% O_2_ for 24 hrs by TEM. White arrows indicate autophagic vesicles. (**B**) Quantitative counting of the number of autophagic vesicles in a 60 μm^2^ square area of each cell in panel A. control 1.594 +/- 1.214, n=32; treated with 1% O_2_ for 24 hrs 8.188 +/- 3.021, n=32, *p<*0.001; (**C**) LAMP1 expression in hypoxic bEnd.3 cells was detected by qRT-PCR. control 1.00 +/- 0.225, n=3; treated with 1% O_2_ for 24 hrs 1.93 +/- 0.337, n=3, *p<*0.05. (**D**) bEnd.3 cells were cultured at 1% O_2_ for the indicated duration. Claudin-5 in hypoxic bEnd.3 cells was detected by Western blot. (**E**) Gray value of Claudin-5 in panel D. control 1.00 +/- 0.118, n=3; treated with 1% O_2_ for 12 hrs 1.104 +/- 0.151, n=3; treated with 1% O_2_ for 24 hrs 0.483 +/- 0.105, n=3, *p<*0.01; treated with 1% O_2_ for 48 hrs 0.439 +/- 0.124, n=3, *p<*0.01. (**F**) Hypoxic bEnd.3 cells were fixed and stained with anti-Claudin-5 and anti-LAMP1 antibodies for confocal imaging using a 63× objective. (**G**) Statistics of Claudin-5 and LAMP1 co-localization ratio in bEnd.3 cells using the Manders' Colocalization Coefficients. control 3.873 +/- 2.149, n=63; treated with 1% O_2_ for 24 hrs 40.86 +/- 6.877, n=94, *p<*0.001. Significance values were denoted as follows: *p<0.05, **p<0.01, and ***p<0.001, with n.s indicating no significance.

As a key protein in vascular endothelial tight junctions, Claudin-5 reduction leads to BBB injury and cerebral edema [43]. The mechanism of Claudin-5 degradation induced by hypoxia in cerebral microvascular endothelial cells was explored. Figs. (**2A** and **B**) show that hypoxia-induced a substantial formation of autophagosomal vesicles in bEnd.3 cells. Concurrently, hypoxia significantly upregulated the lysosomal protein LAMP1 in bEnd.3 cells (Fig. **2C**), indicating hypoxia-induced autophagy in endothelial cells. Prolonged hypoxia exposure significantly downregulated Claudin-5 in bEnd.3 cells (Figs. **2D** and **E**). Similarly, in hypoxia-treated bEnd.3 cells, LAMP1 upregulation was observed alongside a marked reduction in Claudin-5 membrane expression and an increased ratio of Claudin-5 to LAMP1 co-localization (Figs. **2F** and **G**). These results suggest that hypoxia decreases both the total content and membrane expression of Claudin-5 in bEnd.3 cells by inducing autophagic degradation.

### 3.3 HCQ Mitigates Autophagic Degradation of Claudin-5 through the mTOR Pathway

**Fig. (3):**
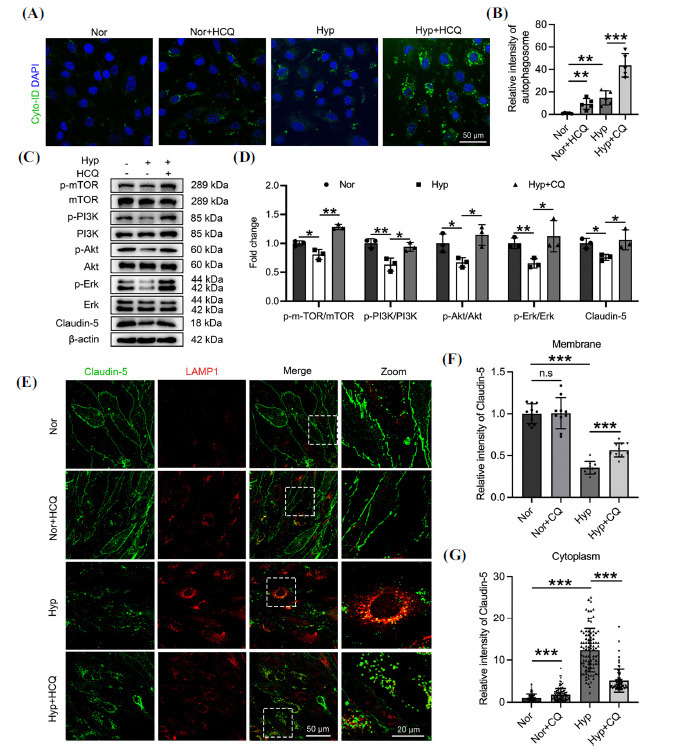
HCQ alleviates hypoxia-induced autophagy and downregulation of Claudin-5 in endothelial cells. bEnd.3 cells were pre-treated with 30 μM HCQ for 2 hrs and co-treated with 1% O_2_ for 24 hrs. (**A**) Autophagic vesicles were labeled using Cyto-ID staining. (**B**) Statistics of Cyto-ID fluorescence intensities in panel A. control 1.00 +/- 0.297, n=5; treatment 30 μM HCQ 11.18 +/- 8.26, n=5, *p<*0.05; treated with 1% O_2_ for 24 hrs 14.78 +/- 6.298, n=5, *p<*0.01; treatment 30 μM HCQ and 1% O_2_ for 24 hrs 43.7 +/- 10.5, n=5, *p<*0.001. (**C**) mTOR, PI3K, Akt, Erk, and their phosphorylation forms were detected by Western blot. (**D**) Statistics of the gray values of p-mTOR/mTOR (control 1.00 +/- 0.045, n=3; treated with 1% O_2_ for 24 hrs 0.81 +/- 0.088, n=3, *p<*0.05; treatment 30 μM HCQ and 1% O_2_ for 24 hrs 1.28 +/- 0.044, n=3, *p<*0.01); p-PI3K/PI3K (control 1.00 +/- 0.079, n=3; treated with 1% O_2_ for 24 hrs 0.63 +/- 0.112, n=3, *p<*0.01; treatment 30 μM HCQ and 1% O_2_ for 24 hrs 0.94 +/- 0.075, n=3, *p<*0.05); p-Akt/Akt (control 1.00 +/- 0.16, n=3; treated with 1% O2 for 24 hrs 0.67 +/- 0.082, n=3, *p<*0.05; treatment 30 μM HCQ and 1% O_2_ for 24 hrs 1.15 +/- 0.177, n=3, *p<*0.05); p-Erk/Erk (control 1.00 +/- 0.093, n=3; treated with 1% O_2_ for 24 hrs 0.66 +/- 0.0825, n=3, *p<*0.01; treatment 30 μM HCQ and 1% O_2_ for 24 hrs 1.12 +/- 0.268, n=3, *p<*0.05); Claudin-5 (control 1.00 +/- 0.086, n=3; treated with 1% O_2_ for 24 hrs 0.76 +/- 0.052, n=3, *p<*0.05; treatment 30 μM HCQ and 1% O_2_ for 24 hrs 1.06 +/- 0.173, n=3, *p<*0.05) in panel C. (**E**) Cells were fixed and stained with anti-Claudin-5 and anti-LAMP1 antibodies for confocal imaging using a 63× objective. (**F** and **G**) Statistics of Claudin-5 fluorescence intensity in the plasma membrane (**F**) (control 1.00 +/- 0.119, n=10; treatment 30 μM HCQ 1.007 +/- 0.185, n=10; treated with 1% O_2_ for 24 hrs 0.355 +/- 0.077, n=10, *p<*0.001; treatment 30 μM HCQ and 1% O_2_ for 24 hrs 0.566 +/- 0.0833, n=10, *p<*0.001) and cytoplasm (**G**) (control 1.00 +/- 0.955, n=133; treatment 30 μM HCQ 1.804 +/- 1.468, n=120, *p<*0.001; treated with 1% O_2_ for 24 hrs 12.35 +/- 5.127, n=93, *p<*0.001; treatment 30 μM HCQ and 1% O_2_ for 24 hrs 5.126 +/- 2.742, n=68, *p<*0.001) of bEnd.3 cells in panel **E**. Significance values were denoted as follows: *p<0.05, **p<0.01, and ***p<0.001, with n.s indicating no significance.

As an autophagy inhibitor, HCQ's effect on autophagic flux in hypoxic bEnd.3 cells was assessed using the Cyto-ID autophagy detection kit. Bright green puncta, indicating autophagic vacuoles [44], were significantly increased in hypoxia-treated bEnd.3 cells, suggesting hypoxia upregulates autophagy in endothelial cells. HCQ further increased autophagosomes in these cells, indicating it blocked the lysosomal degradation of autophagosomes under hypoxia (Figs. **3A** and **B**). The mechanism of HCQ combined with hypoxia on autophagy in bEnd.3 cells was investigated, revealing that hypoxia significantly reduced the phosphorylation of PI3K, mTOR, AKT, and Erk1/2, while HCQ treatment reversed this process (Fig. **3C** and **D**). This indicates that HCQ alleviates hypoxia-induced autophagy *via* the PI3K-Akt-mTOR and Erk signaling pathways. Consistently, HCQ upregulated Claudin-5 membrane expression in hypoxic bEnd.3 cells and significantly reduced its abnormal cytoplasmic accumulation (Figs. **3E**-**3G**, Fig. **S1**). These results demonstrate that HCQ mitigates the autophagic degradation of Claudin-5 by inhibiting the PI3K-Akt-mTOR and Erk signaling pathway, thus preventing hypoxia-induced disruption of endothelial cell TJs.

### 3.4 HCQ Reverses Hypoxia-induced Endothelial Cell Permeability

**Fig. (4):**
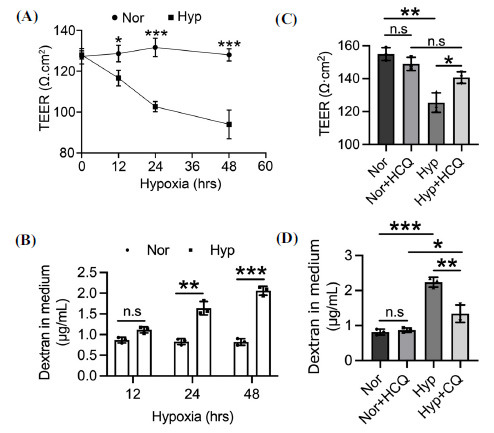
HCQ reduces hypoxia-induced permeability in bEnd.3 cells. (**A** and **B**) bEnd.3 cells were treated with 1% O_2_ for the indicated duration. The resistance of bEnd.3 cells was detected by TEER assay. control 127.33 +/- 3.786, treated with 1% O_2_ for 0 hr 128 +/- 2, n=3; control 128.67 +/- 4.041, treated with 1% O_2_ for 12 hrs 116.67 +/- 3.786, n=3, *p<*0.05; control 131.67 +/- 4.509, treated with 1% O_2_ for 24 hrs 102.67 +/- 2.517, n=3, *p<*0.001; control 128 +/- 3, treated with 1% O_2_ for 48 hrs 94 +/- 7, n=3, *p<*0.001 (**A**). FITC-dextran 40 kDa was added to the transwell and incubated for 30 mins, and the fluorescence intensity of FITC in the lower medium was detected to quantify the concentration. control 0.869 +/- 0.069, treated with 1% O_2_ for 12 hrs 1.114 +/- 0.746, n=3; control 0.831 +/- 0.074, treated with 1% O_2_ for 24 hrs 1.638 +/- 0.163, n=3, *p<*0.01; control 0.823 +/- 0.08, treated with 1% O_2_ for 48 hrs 2.06 +/- 0.107, n=3, *p<*0.001 (**B**). (**C** and **D**) bEnd.3 cells were pre-treated with 30 μM HCQ, then co-treated with 1% O_2_ for 24 hrs. The resistance value of endothelial cells was detected by TEER assay. Control 154.9 +/- 3.933, n=3; treatment 30 μM HCQ 149 +/- 4.038, n=3; treated with 1% O_2_ for 24 hrs 125.4 +/- 5.926, n=3, *p<*0.01; treatment 30 μM HCQ and 1% O_2_ for 24 hrs 140.7 +/- 3.512, n=3, *p<*0.05 (**C**). FITC-dextran 40 kDa was added to the transwell and incubated for 30 mins, and the fluorescence intensity of FITC in the lower medium was detected to quantify the concentration. control 0.814 +/- 0.084, n=3; treatment 30 μM HCQ 0.868 +/- 0.065, n=3; treated with 1% O_2_ for 24 hrs 2.24 +/- 0.14, n=3, *p<*0.001; treatment 30 μM HCQ and 1% O_2_ for 24 hrs 1.338 +/- 0.249, n=3, *p<*0.01 (**D**). Significance values were denoted as follows: *p<0.05, **p<0.01, and ***p<0.001, with n.s indicating no significance.

The effect of HCQ on endothelial cell permeability was further investigated. bEnd.3 cell monolayers were cultured in Transwells to form TJs. The TEER value, inversely correlated with permeability [45, 46], progressively decreased with prolonged hypoxia exposure (Fig. **4A**). Hypoxia also increased the amount of dextran crossing the endothelial cells (Fig. **4B**), indicating heightened permeability. Under normoxic conditions, HCQ did not affect the TEER or dextran permeability of bEnd.3 cells. However, HCQ significantly increased the TEER and decreased dextran permeability in hypoxic bEnd.3 cells (Figs. **4C** and **D**), suggesting HCQ effectively reverses the increased permeability of hypoxic endothelial cells.

### 3.5 Autophagic Degradation of Claudin-5 is Elevated in the Brains of HACE Mice

**Fig. (5):**
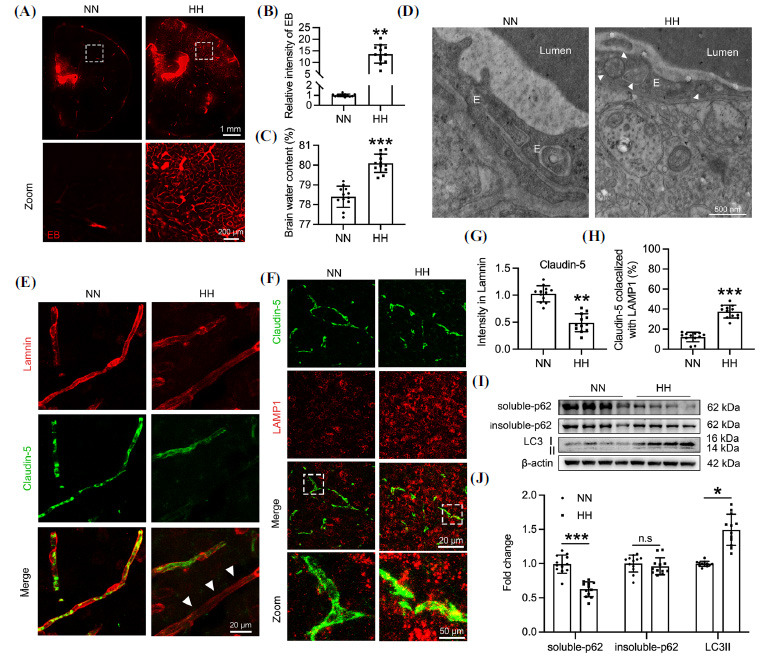
HH exposure enhances autophagy while suppressing Claudin-5 expression in the mouse brain. C57BL/6J mice were exposed to HH (7,000 m above sea level) for 48 hrs. (**A**) Leakage of EB in the cerebral cortex and striatum of mice was observed by confocal microscopy. (**B**) The intensity of EB in panel **A** was quantified. NN 1.00 +/- 0.1269, n=12; treated with HH 13.62 +/- 3.919, n=12, *p<*0.01. (**C**) Brain water content was measured by wet and dry weight. NN 78.4 +/- 0.5388, n=12; treated with HH 80.09 +/- 0.4618, n=12, *p<*0.001. (**D**) The number of autophagic vesicles in the mouse brain was analyzed by TEM. White arrows indicate autophagic vesicles, and the letter E represents endothelial cells. (**E**) Laminin was labeled using immunofluorescence to show vascular basement membranes in brain tissue, and Claudin-5 was co-labeled to show the continuity of TJs. White arrows indicate the discontinuity of Claudin-5. (**F**) Sections of mouse brain tissue were co-labeled with anti-Claudin-5 and anti-LAMP1 antibodies. (**G**) Quantification of Claudin-5 intensity in panel **E**. NN 1.00 +/- 0.1497, n=12; treated with HH 0.489 +/- 0.166, n=12, *p<*0.01. (**H**) The co-localization ratio of Claudin-5 and LAMP1 was analyzed using Manders' Colocalization Coefficients. NN 12.11 +/- 4.772, n=12; treated with HH 37.51 +/- 6.407, n=12, *p<*0.001. (**I**) Soluble and insoluble proteins were extracted from the mouse cerebral cortex, p62 and LC3 were detected by Western blot. (**J**) Quantification of gray values of soluble-p62 (NN 1.00 +/- 0.133, n=12; treated with HH 0.625 +/- 0.109, n=12, *p<*0.001), insoluble-p62 (NN 1.00 +/- 0.124, n=12; treated with HH 0.961 +/- 0.124, n=12), and LC3 (NN 1.00 +/- 0.038, n=12; treated with HH 1.493 +/- 0.228, n=12, *p<*0.05) bands in panel **I**. Significance values were denoted as follows: *p<0.05, **p<0.01, and ***p<0.001, with n.s indicating no significance.

C57BL/6J mice were exposed to an altitude of 7,000 m for 48 hrs to establish a HACE model. The EB leakage significantly increased in the brains of HH mice (Figs. **5A** and **B**), indicating enhanced vascular permeability. Additionally, brain water content markedly increased following HH exposure (Fig. **5C**), consistent with HACE characteristics. Autophagosome numbers in the cerebral cortex's microvascular endothelium of HH mice significantly rose (Fig. **5D**), suggesting HH-induced vascular permeability through upregulated autophagy. Claudin-5 levels in cerebral microvascular endothelium were also assessed. HH exposure significantly impaired Claudin-5 continuity and reduced its amount (Figs. **5E** and **G**). Moreover, the co-localization of Claudin-5 with LAMP1 in cerebral vascular endothelium increased significantly in HH mice, indicating HH-promoted lysosomal degradation of Claudin-5 (Figs. **5F** and **H**). Further examination of brain autophagy showed significant downregulation of soluble p62 in the cerebral cortex of HH-exposed mice, while insoluble p62 levels remained unchanged. The conversion of LC3-I to LC3-II increased, demonstrating upregulated autophagy in brain tissue post-HH exposure (Figs. **5I** and **J**). Collectively, these findings indicate that HH exposure enhances autophagy in the mouse brain, leading to the autophagic degradation of Claudin-5, resulting in endothelial TJ damage and increased BBB permeability, thus inducing HACE.

### 3.6 HCQ Prevents HACE by Inhibiting Autophagic Degradation of Claudin-5

**Fig. (6):**
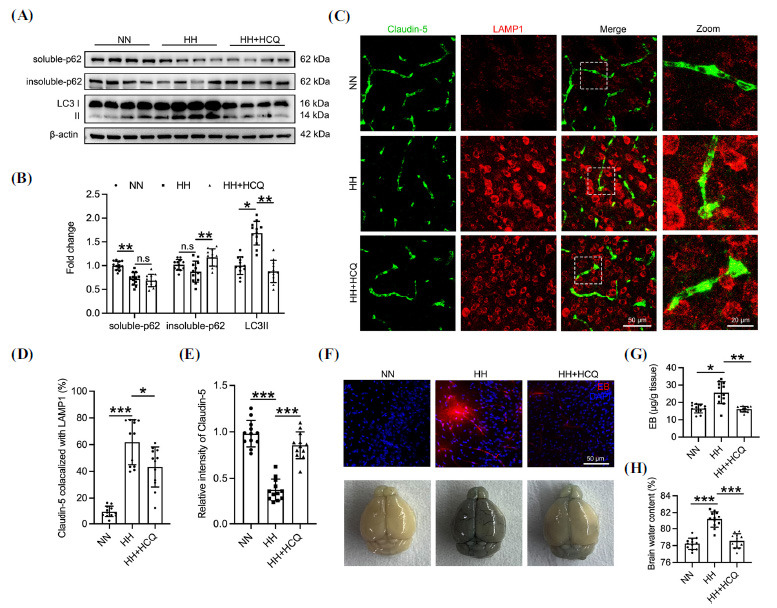
HCQ alleviates autophagic degradation of Claudin-5 and reduces brain water content in HH mice. C57BL/6J mice were subjected to HH exposure (7,000 m above sea level) for 48 hrs after intraperitoneal injection of HCQ (30 mg/kg, 2 injections at 12-hrs intervals). (**A**) Soluble and insoluble proteins were isolated from the cerebral cortex for p62 and LC3 detection by Western blot. (**B**) Quantification of gray values of soluble-p62 (NN 1.00 +/- 0.094, n=12; treated with HH 0.727 +/- 0.132, n=12, *p<*0.01; treated with HH and HCQ 0.687 +/- 0.131, n=12), insoluble-p62 (NN 1.00 +/- 0.114, n=12; treated with HH 0.873 +/- 0.222, n=12; treated with HH and HCQ 1.172 +/- 0.181, n=12, *p<*0.01), and LC3 (NN 1.00 +/- 0.187, n=12; treated with HH 1.683 +/- 0.249, n=12, *p<*0.05; treated with HH and HCQ 0.877 +/- 0.235, n=12, *p<*0.01) bands in panel **A**. (**C**) Brain sections were labeled with anti-Claudin-5 and anti-LAMP1 antibodies. (**D**) Co-localization ratio of Claudin-5 and LAMP1 in panel **C**. NN 9.758 +/- 4.191, n=12; treated with HH 61.86 +/- 16.95, n=12, *p<*0.001; treated with HH and HCQ 43.31 +/- 14.98, n=12, *p<*0.05 (**E**) Quantification of Claudin-5 fluorescence intensity in panel **C**. NN 1.00 +/- 0.1424, n=12; treated with HH 0.3757 +/- 0.116, n=12, *p<*0.001; treated with HH and HCQ 0.8558 +/- 0.1439, n=12, *p<*0.001. (**F**) Mice were injected with EB through the tail vein, circulated for 1 h, and the leakage of EB in brain tissue was observed using confocal microscopy. (**G**) Quantification of residual EB content in brain tissue by colorimetric method. NN 16.46 +/- 2.489, n=12; treated with HH 25.55 +/- 6.311, n=12, *p<*0.05; treated with HH and HCQ 16.08 +/- 1.57, n=12, *p<*0.01. (**H**) Brain water content was measured by wet and dry weight. NN 78.22 +/- 0.6623, n=12; treated with HH 81.14 +/- 0.9617, n=12, *p<*0.001; treated with HH and HCQ 78.56 +/- 0.8344, n=12, *p<*0.001. Significance values were denoted as follows: *p<0.05, **p<0.01, and ***p<0.001, with n.s indicating no significance.

To explore the therapeutic effect of HCQ on HACE, C57BL/6J mice received intraperitoneal injections of HCQ (30 mg/kg, two injections at 12-hrs intervals) and were subsequently exposed to HH conditions (7,000 m altitude) for 48 hrs. HCQ treatment upregulated insoluble p62 and attenuated LC3 in the brains of HH-exposed mice, indicating effective inhibition of HH-induced autophagy by HCQ (Figs. **6A** and **B**). Additionally, HCQ significantly reduced Claudin-5 and LAMP1 co-localization in the cerebral blood vessels of HH mice and upregulated Claudin-5 levels (Figs. **6C**-**E**), suggesting HCQ attenuated HH-induced Claudin-5 degradation. Furthermore, HCQ significantly reduced microvascular leakage in the brains of HH mice (Figs. **6F** and **G**), demonstrating HCQ's efficacy in ameliorating HH-induced BBB permeability increase. HCQ also prevented the rise in brain water content in HH mice (Fig. **6H**), thereby preventing HACE.

## 4 DISCUSSION

In our previous study, we investigated the specific mechanism by which Caveolin-1 mediates endocytosis during the occurrence and progression of HACE. Furthermore, endothelial autophagy facilitates the clearance of aggregated claudin-5 within the cytosol, resulting in BBB disruption and thereby triggering HACE [18]. On this foundation, we delve deeper into the impact of the autophagy inhibitor HCQ on HACE following the inhibition of autophagy, thereby repurposing an established medication. The findings from this study establish a solid basis for subsequent investigations into the therapeutic possibilities of HCQ and the role of autophagy in the pathogenesis of HACE.

Recent studies have demonstrated that environmental stimuli, such as hypoxia, activate autophagy [47]. The regulation of autophagy is crucial in the pathophysiological processes linked to HACE. As illustrated in Fig. (**5D**), it is evident that autophagy’s role under hypoxic conditions extends beyond endothelial cells, affecting a diverse array of cell types. Furthermore, Choi *et al*. observed that excessive autophagy activation in neurons and microglia, resulting from hypoxic-ischemic brain injury, led to reduced neuronal density and axonal demyelination, thereby causing neuronal damage and exacerbating cognitive, learning, and memory impairments [48]. Additionally, Wang *et al*. reported that hypoxia stimulated microglia to release proinflammatory factors, promoting their recruitment to blood vessels and resulting in endothelial cell damage and disruption of BBB permeability [37]. Damri *et al*. discovered that hypoxia-induced ROS upregulation triggered excessive autophagy activation in neurons, leading to abnormal neuronal death [49]. Consequently, it is hypothesized that exposure to HH may initiate autophagy activation in various brain cells, including microglia and neurons, thereby contributing to the onset of HACE. This implies that HCQ may exert its protective effects on multiple central nervous system cells by modulating their functions, thereby preventing HACE.

Specifically, the ability of HCQ to alter the pH within lysosomes and inhibit the activity of acidic proteases may play a crucial role in this process. HCQ is a weak base that easily crosses cell membranes and accumulates in acidic subcellular compartments such as lysosomes and endosomes, where they remain trapped in a protonated state. This leads to a pH increase in lysosomes from 4 to 6, causing inhibition of acidic proteases and other enzymes within the endolysosomal compartments [50]. Autophagy inhibitors have shown promise in preclinical studies by inhibiting lysosomal acidification and preventing autophagosome degradation [51]. In our prior research, we uncovered that Caveolin-1 promotes the endocytosis of the tight junction protein Claudin-5, which is then transported to autophagolysosomes *via* endosomes. The autophagic degradation of Claudin-5 disrupts vascular endothelial tight junctions, thereby heightening endothelial cell permeability [18]. However, the administration of HCQ effectively alleviates autophagy, reverses the autophagic degradation of Claudin-5, and may promote its recycling back to the membrane under the influence of Caveolin-1. This process likely enhances the integrity of the BBB and alleviates HACE. Thus, this implies that although HCQ accumulates within lysosomes, the data suggests that there are also effects taking place outside the lysosomal compartment. Moreover, these findings underscore the potential of HCQ as a therapeutic agent in conditions characterized by compromised BBB function. Further investigation into its mechanisms of action could unveil additional benefits in the management of various neurological disorders associated with autophagy dysregulation.

Currently, HCQ, though not yet directly employed for treating autophagy-related disorders, has demonstrated efficacy in modulating autophagy processes. This modulation enhances the effectiveness of albumin-bound paclitaxel against pancreatic cancer cells [52]. In the therapy for Corona Virus Disease 2019, HCQ has exhibited inhibitory effects on SARS-CoV-2 infection by selectively suppressing autophagy molecules [53]. Additionally, HCQ alleviates symptoms in patients with systemic lupus erythematosus by regulating the Th17/Treg immune balance [54]. Focusing on the pivotal PI3K/AKT/mTOR pathway, HCQ's regulatory role suggests its potential to boost tumor chemosensitivity and treat various conditions. This pathway is crucial in tumor initiation, progression, and diseases such as osteoarthritis, with HCQ intervention potentially leading to positive therapeutic outcomes [55-57]. Moreover, inhibiting the PI3K-AKT/mTOR pathway has shown benefits in autism treatment [58]. HCQ also reduces endothelial inflammation by blocking inflammatory pathways such as NF-κB, p38, and JNK, offering a novel approach to treating inflammatory vascular diseases [59]. Furthermore, HCQ improves endothelial function, activates insulin signaling pathways, inhibits fat generation and autophagy, and exerts comprehensive effects against hyperglycemia and hyperlipidemia. It promotes cardiac protection, manages hypertension, and combats obesity by reducing inflammation and oxidative stress [60]. Hence, with its extensive biological activities and multi-target mechanism, HCQ holds significant potential as a candidate drug for various clinical treatments.

In this study, it was found that HCQ alleviated autophagy in bEnd.3 cells *via* the PI3K-Akt-mTOR and Erk pathway, reducing hypoxia-induced Claudin-5 down-regulation. However, the downregulation of TJs can also be influenced by factors beyond autophagy, such as inflammation [61, 62], transendothelial migration of inflammatory cells [63], apoptosis [64], and reorganization of the actin cytoskeleton [65]. Hypoxia, as is the main stress in the cell culture and animal model, is well known to cause substantial changes to the cytoskeleton, in particular, the actin filament dynamics. Studies have demonstrated that actomyosin contractility plays a crucial role in mediating hypoxia-induced barrier dysfunction by modulating junctional Claudin-5. Hence, the deterioration of tight junctions could be directly caused by actin reorganization [66]. Additionally, the internalization of other TJs, such as Occludin and Claudin-5, has been linked to caveolin shuttling [18, 67]. Hypoxia-induced redistribution of actin appears to be mediated by components downstream of MAPK p38, which is activated in response to hypoxia in lung endothelial cells [68]. Hypoxia, in particular, reoxygenation, is tied to a massive production of oxygen radicals, leading to many degenerative processes in the cell, and also strongly affects the mTOR pathway [69-71]. The mTOR pathway consists of multiple components regulated by phosphorylation or dephosphorylation. Oxidative stress often compromises phosphates (active center cysteines are oxidized) and hence leads to an apparent increase in phosphorylation, thereby altering the actin distribution in response to various stimuli [72]. These additional mechanisms highlight HCQ's broad regulatory role in preventing HACE, underscoring its potential to intervene at multiple levels to maintain BBB integrity. The precise mechanisms underlying these processes warrant further in-depth investigation.

TJs are essential components of the cellular barrier that maintain the integrity of epithelial and endothelial tissues. These junctions, primarily composed of Claudins, Occludins, and junctional adhesion molecules, prevent the infiltration of blood-borne molecules into tissues through intercellular gaps, thereby stabilizing the internal environment [73]. Claudin-5 is a crucial component of TJs that maintains barrier integrity and regulates BBB permeability [74]. Campbell *et al*. discovered that targeted inhibition of Claudin-5 partially induced focal brain edema in mice [75]. Other studies have demonstrated that DL-3-n-butylphthalide-mediated upregulation of Claudin-5 ameliorates BBB disruption following ischemic injury [76]. This research revealed that hypoxia increases the permeability of brain microvascular endothelial cells and facilitates the development of HACE through Claudin-5 downregulation. Furthermore, HCQ effectively mitigated the increase in brain water content and BBB permeability after HH exposure by inhibiting Claudin-5 degradation *via* the autophagic lysosomal pathway, thereby delaying HACE progression. Considering that TJs consist of various proteins and that hypoxia downregulates multiple TJ components, HCQ's protective effect on BBB integrity may arise from its ability to mitigate the downregulation of these multiple TJ proteins.

The increasing number of individuals exposed to high altitudes highlights the urgent need for safe and effective medications for the treatment of HACE. This study has definitively established that Claudin-5 autophagic degradation is a predominant mechanism of tight junction disruption. Furthermore, HCQ has been identified to inhibit the binding of autophagy to lysosomes, thereby preventing the intracellular degradation of Claudin-5. The partial ameliorative effect of HCQ on HACE suggests its potential utility in the development of targeted therapeutic agents for this condition. Consequently, the effective inhibition of autophagic degradation emerges as a promising therapeutic strategy for both the prevention and treatment of HACE. Although there are currently no studies reporting the application of HCQ in HACE treatment, future research can leverage our findings to advance clinical interventions.

However, during the *in vitro* experiments conducted in this study, the utilization of bEnd.3 monolayer endothelial cells omitted the interactions of the neurovascular unit, including pericytes and astrocytes, thereby failing to fully replicate the intricate microenvironment of the BBB *in vivo*. Furthermore, the animal models exclusively employed mice, whereas the pathological progression of human HACE, such as the rate of cerebral edema formation, may significantly differ from that observed in rodents, leading to insufficient physiological relevance of the model system. Consequently, to more accurately simulate and investigate HACE, future research should prioritize the development of more complex and physiologically pertinent model systems. This approach is expected to yield more precise and reliable experimental outcomes, thereby facilitating the translation of basic research into clinical practice and offering innovative therapeutic strategies for high-altitude diseases and other disorders related to BBB dysfunction.

## CONCLUSION

By inhibiting mTOR-mediated autophagy, HCQ effectively mitigates the degradation of Claudin-5 in endothelial cells, thereby maintaining the integrity of the BBB. Furthermore, HCQ significantly alleviates the increased BBB permeability induced by HH, efficiently preventing the development of HACE. These findings position HCQ as a promising therapeutic candidate for HACE treatment.

The current study provides compelling evidence that autophagy plays a pivotal role in the pathogenesis of HACE. The outcomes of this study lay the groundwork for further research on the role of autophagy in the pathogenesis of HACE and the therapeutic potential of HCQ. Future studies could delve into the mechanisms underlying the protective effects of HCQ on endothelial cells and the BBB, as well as assess the efficacy and safety of HCQ in clinical trials for HACE treatment. Additionally, exploring other autophagy inhibitors and their potential in managing HACE is a worthwhile pursuit. Overall, the findings of this study provide new insights into the treatment of HACE and emphasize the strategic importance of targeting autophagy as a viable therapeutic option for this condition.

## AUTHORS’ CONTRIBUTIONS

The authors confirm their contribution to the paper as follows: LZ and XTW conceived and designed the experiments. YX, BLW, and ZW performed the experiments and analyzed the data. YX wrote the initial draft of the manuscript. ZWW, DZW, and WPY designed and illustrated the scheme. All authors reviewed the results and approved the final version of the manuscript.

## Data Availability

All additional data supporting the findings of this study are available from the corresponding authors upon reasonable request.

## LIST OF ABBREVIATIONS

BBB: Blood-brain Barrier
EB: Evans Blue
HACE: High-altitude Cerebral Edema
HCQ: Hydroxychloroquine
HH: Hypobaric Hypoxia
NN: Normobaric Normoxia
TEER: Transendothelial Electrical Resistance
TEM: Transmission Electron Microscopy
TJs: Tight Junction Proteins
