# Supplementary material for: Hydroxychloroquine Prevents High-altitude Cerebral Edema by Inhibiting Endothelial Claudin-5 Autophagic Degradation
Source: Curr Neuropharmacol. 2025 May 9;24(3):404–18. doi: 10.2174/011570159X371235250417051313 (PMC13084762; doi:10.2174/011570159X371235250417051313)
Supplement: Supplementary file 1 [file CN-24-3-404_SD1.pdf]

Supplementary Material

Hydroxychloroquine Prevents High-altitude Cerebral Edema by Inhibiting Endothelial Claudin-5 Autophagic Degradation

Yan Xue[1,2,#], Baolan Wan[1,#], Zhen Wang[3,#], Zhiwei Wang[3], Dongzhi Wang[3], Wanping Yang[2], Xueting Wang[2,*] and Li Zhu[2,*]

[1]Nantong Health College of Jiangsu Province, Nantong 226010, China; [2]Institute of Special Environmental Medicine, Co-Innovation Center of Neuroregeneration, Nantong 226019, China; [3]Department of Hepatobiliary and Pancreatic Surgery, Affiliated Hospital of Nantong University, Nantong 226006, China

1570-159X/26 $65.00+.00 **© 2026 Bentham Science Publishers**

Fig. S1 HCQ inhibits autophagic degradation of Claudin-5. Western blot analysis revealed that hypoxia treatment led to a decrease in Claudin-5 protein levels in both total and membrane proteins of endothelial cells. Notably, blocking hypoxia-induced autophagy with HCQ effectively inhibited the degradation of total and cytoplasmic Claudin-5 proteins. Conversely, rapamycin stimulation further exacerbated the loss of Claudin-5 in both membrane and total proteins (Fig. S1A and B). These results indicate that inhibiting autophagy prevents hypoxia-induced intracellular degradation of endothelial Claudin-5 proteins and mitigates the loss of Claudin-5 in membrane proteins.

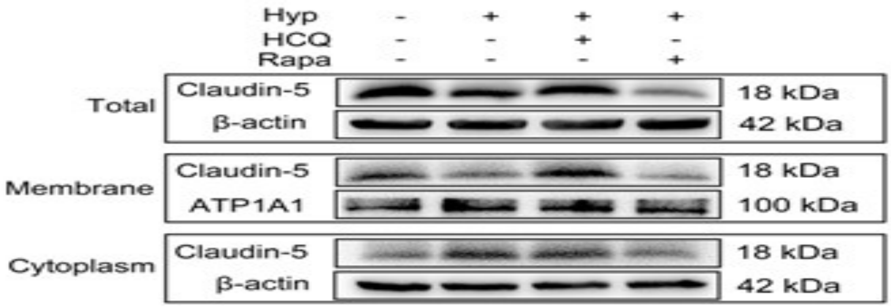

(A)

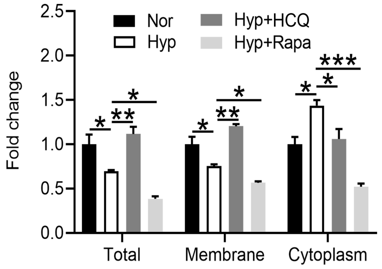

(B)

Fig. (S1). Blockade of autophagy inhibits hypoxia-induced degradation of endothelial Claudin-5. (**A**) Endothelial cells were collected to extract total, membrane, and cytoplasmic proteins, and Claudin-5 protein expression was detected by Western blot. (**B**) Gray values of Claudin-5 bands were quantified in panel A. $n = 3$. * $P < 0.05$, ** $P < 0.01$, and *** $P < 0.001$.
